# KUNO-Kids birth cohort study: rationale, design, and cohort description

**DOI:** 10.1186/s40348-018-0088-z

**Published:** 2019-01-09

**Authors:** Susanne Brandstetter, Antoaneta A. Toncheva, Jakob Niggel, Christine Wolff, Silvia Gran, Birgit Seelbach-Göbel, Christian Apfelbacher, Michael Melter, Michael Kabesch, the KUNO-Kids study group, Petra Arndt, Andrea Baessler, Mark Berneburg, Wolfgang Buchalla, Sara Fill Malfertheiner, André Gessner, Iris Heid, Sebastian Kerzel, Michael Koller, Michael Leitzmann, David Rothfuß, Wolfgang Rösch, Hugo Segerer, Bernhard H. F. Weber, Stephan Weidinger

**Affiliations:** 1 University Children’s Hospital Regensburg (KUNO-Clinics), University of Regensburg, Clinic St. Hedwig, Steinmetzstr. 1-3, 93049 Regensburg, Germany; 2 Clinic of Obstetrics and Gynecology St. Hedwig, University of Regensburg, Regensburg, Germany; 3 Medical Sociology, Institute of Epidemiology and Preventive Medicine, University of Regensburg, Regensburg, Germany

**Keywords:** Birth cohort, Child health, Study design, Participation, Omics

## Abstract

**Background:**

Birth cohort studies can contribute substantially to the understanding of health and disease — in childhood and over the life course. The KUNO-Kids birth cohort study was established to investigate various aspects of child health, using novel omics technologies in a systems medicine approach.

**Results:**

After 3 years of recruitment, 2515 infants and their families have joined the study. Parents with higher education are overrepresented as in many other birth cohorts and are more likely to complete follow-up assessments via self-report questionnaires. The vast majority of participants consented to clinical examinations of their child and to the non-invasive collection of diverse biosamples, which were processed specifically for their integrated use in omics technology covering genomics, epigenomics, transcriptomics, metabolomics, and microbiome analyses of the skin, oral cavity, and stool.

**Conclusions:**

The data and diverse biomaterial collected in the KUNO-Kids birth cohort study will provide extensive opportunities for investigating child health and its determinants in a holistic approach. The combination of a broad range of research questions in one study will allow for a cost-effective use of biomaterial and omics results and for a comprehensive analysis of biological and social determinants of health and disease. Aiming for low attrition and ensuring participants’ long-term commitment will be crucial to fully exploit the potential of the study.

## Background

Birth cohort studies have contributed significantly to advancing medical knowledge about childhood and adult health. A particular strength of such studies is their potential to identify mechanisms of health and disease over the life course through prospective follow-up. Although bias and confounding are serious problems in observational research, associations between exposures and outcomes can be established [1], which might even provide evidence for causal pathways. Links between exposures from early childhood to subsequent disease outcomes in adulthood are of particular relevance [2]. The number of available birth cohorts is substantial (birthcohorts.net [3, 4, 5]), but findings cannot always be readily transferred from one population to another because environmental, socio-economic, and cultural factors differ greatly between populations. Due to rapid lifestyle, societal, and environmental changes in many societies over the past decades, birth cohorts can become outdated within just a few years, no longer being applicable to the present generation. To consider recent developments within the population and make health decisions on the personal and population level based on knowledge rather than on assumptions, the availability of current data gained through state of the art assessment is crucial.

Thus, in a contemporary birth cohort study, recent socio-cultural developments in Germany ought to be reflected, including the shifting roles of motherhood and fatherhood, facilitated by financial support for parental leave [6, 7], increasing availability of early child care [8], rising birth rates [9], and widespread digital technology in work and home environments [10, 11]. Furthermore, medical knowledge has progressed significantly in many fields, leading to new health promotion and prevention programs. Finally, novel scientific methods and research paradigms have emerged rapidly in recent years driven by a big leap in omics technologies allowing measurements of countless molecular parameters describing the genome, the epigenome, the transcriptome, and the metabolome as well as the microbiome of an individual with an unprecedented abundance of information. On the other hand, a vast amount of environmental influences (exposome) can now be measured with novel tools such as satellite data, personal tracking devices, and advanced biochemical screening tools [12]. Taken together, an integrated systems biology approach to better understand the development of health and disease has now become feasible for birth cohort studies at a level previously not possible.

Against this background, the KUNO-Kids birth cohort study was established with three major objectives: (a) to contribute to the understanding of current child health using novel omics technologies in a systems medicine approach, (b) to identify novel modifiable factors of child health and opportunities for prevention, and (c) to provide a platform for investigating the feasibility and effectiveness of targeted interventions. Here, we provide an overview of the rationale and design of the KUNO-Kids birth cohort study, describing the characteristics of study participants after 3 years of recruitment and the procedures for sampling and processing biomaterial for omics analysis, as first analyses based on the current dataset from this study will soon be available.

## Methods

### Design

**Fig. 1:**
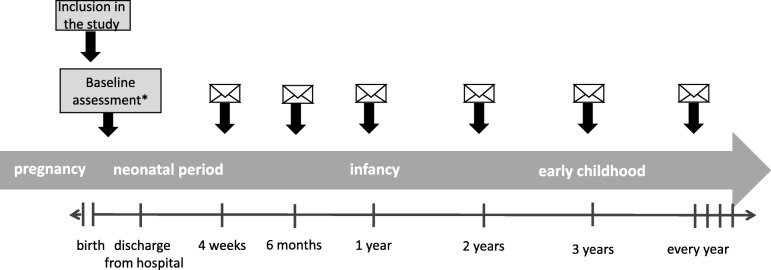
Study design of the KUNO-Kids birth cohort study. The baseline assessment includes clinical examinations, collection of biosamples, and retrospective assessment of exposures occurring before or during pregnancy using standardized interviews and self-report questionnaires

KUNO-Kids is a multi-purpose birth cohort study, which aims at investigating a wide range of exposures and outcomes. Data are collected not only from the index child (or children in the case of multiples), but also from his/her family (including mother, father, and siblings). The inclusion in the study starts immediately after birth of the index child. The baseline assessment takes place during the short hospital stay of mother and child directly after birth. Regular follow-up assessments are conducted when the index child is 4 weeks old, 6 months old, and at each birthday. Figure 1 provides an overview of the study design and the time points of data collection.

### Catchment area, setting, and recruitment of study participants

The study is based in Eastern Bavaria (Regensburg, Germany). The catchment area is characterized by the city of Regensburg (164,000 inhabitants, among them 32,000 university students) and its predominantly rural adjacent regions. Rates of unemployment are among the lowest in Germany [13], and the population size is currently rising [14]. The clinic St. Hedwig is a modern children’s and women's hospital affiliated with Regensburg University where about two thirds of the children from the region are born (approximately 3000 births per year). Being a tertiary perinatal center, the proportion of high-risk births is large.

After childbirth, mothers at the clinic St. Hedwig are approached by trained study personnel and are informed about the study aims and procedures. Mothers are eligible for enrolment in the study if they are 18 years of age or older and if they are able to provide informed consent (i.e., basic German language skills for the comprehension of study procedures are required). There are no further exclusion criteria. Only one child (or twin pair or triplet) per family is included in the study with the index birth event which initiated recruitment. Characteristics of siblings are assessed by questionnaires in the course of the study. Reasons for non-participation are assessed in mothers who do not consent to participate in the study. Recruitment began in June, 2015, and will be continued for at least five more years.

### Sample size and participant flow

Within this timeframe, the study is designed to enroll at least 5000 and not more than 10,000 newborns and their families. Loss-to-follow-up was assumed to be substantial, particularly for the time period between inclusion at birth and the first follow-up. Therefore, a large number of study participants were considered necessary to provide adequate sample size for a majority of the proposed research questions. However, an a priori sample size estimation was not considered useful as the KUNO-Kids birth cohort is intended to cover a wide array of research questions which relate to different outcomes at various time points, thus leading to a large range of power estimates.

### Measurements and data collection

Data are collected on the level of the child, his/her mother, father, and siblings. Most variables of interest will be assessed repeatedly over time; Fig. 1 depicts the measurement points of the study. Exposures before or during pregnancy are assessed retrospectively. Table 1 provides an overview of the domains covered by the study, including the most important exposures and outcomes as well as their respective assessment methods. In addition to general health outcomes of the child, a range of disease-specific outcomes is assessed (Table 2). Exposures and outcomes were selected to cover a broad range of health aspects in childhood based on results from previous studies such as Ulm Spatz [15, 16], KiGGS [17], ISAAC [18], and only recently identified influences on health such as exposure to digital media. Operationalization and assessment methods were selected to allow for joint analyses of data with two existing birth cohort studies from Germany (Ulm SPATZ [15, 16] and LIFE child in Leipzig [19]), and whenever available, validated questions and measurement instruments were used (see Table 2).

**Table 1:** Important domains, level of assessment, and assessment methods

Domains	Child	Mother	Father	Sibling(s)	Assessment
Socio-demographic information: age, living circumstances, marital status, employment, education, subjective social status (McArthur Scale [35]), health insurance		x	x		Interview; self-report
Pregnancy and birth: environmental exposure, medication, lifestyle behaviors, mode of delivery, duration of pregnancy, complications		x			Interview; self-report; medical records
Medical history: chronic conditions/diseases, hereditary diseases		x	x	x	Interview; self-/proxy-report
Health behaviors: (breast) feeding, nutrition, physical activity, sleep (CSHQ [36], PSQI [37]), smoking, alcohol consumption, sun bathing and sun protection, teeth brushing, media consumption	(x)	x	x		Self-/proxy-report
Home environment: exposure to allergens, measures to control allergens, smoking, urbanity	x				Proxy-report
Psychosocial constructs: stress (EBI [38]), social support (F-SozU [39]), anxiety and depression (PHQ-D [40]), health-related quality of life (SF-12 [41]), health literacy (HLS-EU [35])		x	x		Self-report
Utilization of medical and non-medical services: health check-ups, vaccinations, ambulatory and stationary health care, medication, early interventions, counseling	x	x			Self-/proxy-report
General health outcomes: health status, health-related quality of life, physical development, mental development, obesity, accidents; specific diseases and syndromes^a^	x				Clinical examination; proxy-report

*CSHQ* Childhood Sleep Habits Questionnaire, *EBI* Parental Stress Index, *F-SozU* Social Support Questionnaire, *HLS-EU* European Health Literacy Survey, *PSQI* Pittsburgh Sleep Quality Index, *SF-12* Short Form Health Survey

^a^For a comprehensive description of disease-specific outcomes considered in the KUNO-Kids birth cohort study, please see Table 2

**Table 2:** Disease-specific outcomes currently investigated

	Diseases/syndromes
Allergic diseases	Food allergy, asthma, hayfever, atopic dermatitis
Cardiac diseases	Long QT syndrome
Dermatological diseases	Sequelae of sunburns, atopic dermatitis, erythema toxicum neonatorum, skin lesions
Gastroenterological diseases	Celiac disease, infantile colic, inflammatory bowel disease
Neurological diseases	CNS infection, febrile seizure
Oral health	Tooth decay
Urological disorders	Cryptorchidism, hypospadias, enuresis, urinary tract infections, urolithiasis, hematuria, biliary atresia

### Interview and questionnaires

After inclusion in the study, a personal interview is conducted with the mother. All interviewers are medical doctoral students trained in study procedures and interview techniques according to a standardized training protocol. During follow-up, data are collected by self-report postal questionnaires, which include sections for the study child, mother, father, and if applicable, for the sibling(s) of the study child.

### Clinical and anthropometric assessments

Children participating in the study are assessed in the clinic after birth by a pediatrician according to a predefined standardized clinical procedure (based on an extended baby health check-up (called “U2-examination” in Germany) [20]) between 24 and 72 h after birth. The physical examination includes a whole body status, including the head, skin, chest, and abdominal organs, genitals, musculoskeletal and nervous systems as wells as the sensory organs. Anthropometric measurements of body length, weight, and head circumference are carried out. In addition, a standard 12-channel ECG is recorded and the oxygen saturation is assessed by pulse oximetry in order to screen for critical congenital heart defects.

### Sampling of biomaterials

Biological samples of the child are collected at baseline. Because the study was specifically designed to collect biomaterials for omics approaches, a wide variety of material is collected according to predefined sampling procedures, taking into account (a) the limited amount of biosamples that can be collected from newborns and (b) the adherence to the principle of non-invasiveness in children. Therefore, neonatal blood was collected as cord blood, stool and urine were collected non-invasively by catching methods, and skin and buccal swabs were acquired. The time period for collecting samples such as cord blood is very restricted, and mothers’ informed consent is gathered only after delivery. Therefore, cord blood samples are stored from all newborns until the parents have decided for or against participation in the study. If no informed consent to study participation and biosampling is present, stored samples are destroyed. Only samples of study participants are kept, stored, and worked up.

For all samples, digital bioprotocols for tracing sampling procedures, storage, and sample work up were developed. An overview of the sampling methods and subsequent analyses is provided in Table 3. The collection of samples and the pre-analytic procedures are performed according to standard operating procedures (SOPs) available from the authors upon request.

**Table 3:** Biological sampling methods and analyses

Specimen	Purpose	Downstream applications
Cord blood—EDTA	Genomic DNA isolation	Genotyping, sequencing, epigenetics (methylation) analyses
Cord blood—PAXgene	RNA and miRNA isolation	Transcriptomics
Cord blood—SSR (Clot Activator Tube)	Serum separation	Protein measurements: cytokines, allergens; metabolomics (NMR, LC-MS)
Cord blood—Li-Heparin	Plasma separation	Protein measurements: cytokines, allergens; metabolomics (NMR, LC-MS)
Stool	Bacterial DNA isolation	Microbiome analyses
Urine		Metabolomics (NMR, LC-MS)
Skin swabs—cheek, elbow, forearm	Bacterial DNA isolation	Microbiome analyses
Buccal swabs	Genomic DNA isolation	Genotyping, sequencing, epigenetics (methylation) analyses
Buccal swabs	RNA and miRNA isolation	Transcriptomics
Hair		Toxicology analyses
Gingival smears	Bacterial DNA isolation	Microbiome analyses

Note: All biosamples are collected from the child at baseline

### Data management and protection

Standardized interviews, questionnaires, and clinical assessment documents as well as biosample protocols were designed using QNOME —a novel software tool specifically developed for biomedical studies, which allows designing customized case report forms and questionnaires from a large range of available items and pre-existing questionnaires. Questionnaires are available as e-paper documents and/or online tools linking the data directly to an automatically generated multidimensional SQL database (available upon request by the authors). All study data are pseudonymized and stored on protected servers. Personal data (name, date of birth, address) are never linked to study data. Medical data and data from questionnaires and biosample ID tracking numbers, as well as results from bioanalyses, are stored separately and can only be linked using protected algorithms.

### Ethics and consent

The study was approved by the Ethics Committee of the University of Regensburg (file number: 14-101-0347). All participating parents provide written informed consent. The consent procedure which reflects all data sources and assessment methods is continuous over the course of the study so that at each follow-up, participants are asked if they will participate in the next follow-up. Furthermore, it provides the option of consenting to certain elements of the study while refusing consent to others (e.g., consenting to complete self-report questionnaires, but declining the sampling of specific biomaterials). The participants have the right to withdraw from the study at any time.

## Results

### Participation and response

After 3 years of recruitment, 2515 infants and their families have joined the study. In addition to the general consent, which is a requirement for study participation, the vast majority of participating mothers also provided specific consent to additional study procedures and analyses: 99% agreed to additional examinations of the infant (e.g., ECG, skin swabs), to the collection and analysis of biological samples, and to sharing data or biological samples with external researchers. Ninety-eight percent of participants consented to be re-contacted by the study team, and 97% agreed to the analysis of genetic information.

Coverage of recruitment within the clinic St. Hedwig, participation rates, and reasons for non-participation were investigated in detail for a 2.5-month time period between December 2017 and February 2018. Of all mothers giving birth in the clinic (*N* = 638) during this period, 73% (*N* = 465) were approached by study personnel and informed about the study. Reasons for failure to approach mothers were (a) acute and critical illness of mother or child, (b) discharge from hospital immediately after birth, or (c) organizational and health care-related issues such as mothers not available due to repeated absence from their hospital room, constant visitors, or necessary clinical examinations and therapies.

Of the mothers who were approached, 33% (*N* = 154) agreed to participate in the study, 64% (*N* = 291) could not be included, and 3% (*N* = 20) met the exclusion criteria (minor; mother already participating in the study with other child). The most frequent reasons for non-participation were insufficient German language skills (31%), mothers’ perception that the study procedures are associated with too much effort (29%), and lack of interest (15%). Other reasons were personal or infant’s ill health (5%) or reluctance to delay being discharged from hospital due to the study (5%).

Table 4 provides an overview of the response rates at various follow-up measurements during the child’s first year of life. The largest study dropout occurs between baseline and the 4-week follow-up (36%); at the 1-year follow-up, the response rate is about 50%.

**Table 4:** Response rates for different follow-up time points after 3 years of recruitment

Time point	Percentage	Number
Baseline	99^a^	2492 (out of 2515)
4-week follow-up	64^b^	1618 (out of 2515)
6-month follow-up	60^b^	1260 (out of 2114)
1-year follow-up	49^b^	784 (out of 1588)

^a^100% (*N* = 2515) refers to all participants who consented to participate in the study

^b^100% refer to those participants who reached the respective follow-up time point

### Sample characteristics

Socio-demographic characteristics of children, mothers, and fathers are presented in Table 5. Mothers’ mean age is 33.9 years (standard deviation (SD) = 4.6), and 56% of the mothers gave birth for the first time. The mean educational level is high in both mothers and fathers, with more than half of participants having achieved a school leaving certificate that qualifies for university entrance. Nearly all fathers and more than half of mothers were employed full-time before the birth of their child. Eighty-five percent of mothers and 86% of fathers were born in Germany.

**Table 5:** Characteristics of included infants and their parents after 3 years of recruitment, separately for all study participants and for respondents at 1-year follow-up

	Participants at baseline (*N* = 2492)		Respondents at 1-year follow-up (*N* = 784)^b^	
Infants				
Sex (female) *N* (%)	2492	1223 (49.1)	784	393 (50.1)
Weight at birth (g) *M* (SD)	2479	3352 (507)	776	3343 (503)
Length at birth (cm) *M* (SD)	2481	51 (2.6)	777	51.4 (2.6)
Duration of pregnancy (weeks) *M* (SD)	2465	39.5 (1.6)	774	39.6 (1.6)
One or more older siblings *N* (%)	2475	1091 (44.0)	775	304 (39.2)
Mothers				
Maternal age (years) *M* (SD)	2462	33.9 (4.6)	773	34.8 (4.2)
Maternal marital status	2447		766	
Married, living together with husband *N* (%)		1919 (78.4)		616 (80.4)
Unmarried, living together with partner (%)		468 (19.1)		136 (17.8)
Unmarried, without partner *N* (%)		32 (1.3)		8 (1.0)
Divorced *N* (%)		26 (1.1)		6 (0.8)
Widowed *N* (%)		2 (0.1)		0
Maternal education	2437		766	
School leaving certificate after less than 10 years of schooling *N* (%)		254 (10.4)		54 (7.0)
School leaving certificate after 10 years of schooling *N* (%)		778 (31.9)		272 (35.5)
University entrance level *N* (%)		1369 (56.2)		498 (65.0)
Other school leaving certificate *N* (%)		16 (0.7)		1 (0.1)
No school leaving certificate *N* (%)		20 (0.8)		4 (0.5)
Maternal employment before birth	2426		760	
Full-time employed *N* (%)		1315 (54.2)		466 (61.3)
Part-time employed *N* (%)		649 (26.8)		193 (25.4)
Marginally/not regularly employed *N* (%)		82 (3.4)		18 (2.4)
Maternal leave, housewife *N* (%)		243 (10.0)		56 (7.4)
Pupil, student *N* (%)		37 (1.5)		16 (2.1)
Seeking for employment *N* (%)		21 (0.9)		2 (0.3)
Other *N* (%)		79 (3.3)		9 (1.2)
Born in Germany *N* (%)	2449	2075 (84.7)	768	690 (89.8)
Nationality	2448		768	
German *N* (%)		2195 (89.7)		719 (93.6)
Other *N* (%)		197 (8.1)		36 (4.7)
German and other *N* (%)		65 (2.3)		13 (1.7)
Fathers				
Paternal education	1412^a^		643	
School leaving certificate after less than 10 years of schooling *N* (%)		234 (16.6)		105 (16.4)
School leaving certificate after 10 years of schooling *N* (%)		304 (21.5)		126 (19.4)
University entrance level *N* (%)		843 (59.7)		401 (62.4)
Other school leaving certificate *N* (%)		15 (1.1)		5 (0.8)
No school leaving certificate *N* (%)		16 (1.1)		6 (0.9)
Paternal employment	1486^a^		676	
Full-time employed *N* (%)		1367 (92.0)		622 (92.0)
Part-time employed *N* (%)		43 (2.9)		24 (3.6)
Marginally/not regularly employed *N* (%)		8 (0.5)		3 (0.4)
Paternal leave, househusband *N* (%)		24 (1.6)		10 (1.5)
Pupil, student *N* (%)		22 (1.5)		8 (1.9)
Seeking for employment *N* (%)		8 (0.5)		4 (0.6)
Other *N* (%)		14 (0.9)		6 (0.9)
Born in Germany *N* (%)	2427	2080 (85.7)	762	701 (92.0)
Nationality	1457^a^		658	
German *N* (%)		1360 (93.3)		619 (94.1)
Other *N* (%)		74 (5.1)		27 (4.1)
German and other *N* (%)		23 (1.6)		12 (1.8)

Notes: *M* mean, *SD* standard deviation, *N* number of observations

^a^Low total *N*s as this information was assessed not at baseline, but at 4-week follow-up

^b^Out of 1588 participants who already reached the 1-year follow-up time point

Study participants who returned the 1-year follow-up questionnaire differed from the baseline sample with regard to socio-demographic characteristics. Mothers with only one child, those with an older age or a higher education level, those with full-time employment before the birth of their child, and those without a migration background were more likely to have completed the 1-year follow-up questionnaire than their respective counterparts (Table 5).

## Discussion

The KUNO-Kids study is a holistic “next generation” birth cohort which combines a broad phenotyping approach with a wide spectrum of omics technologies. The involvement of experts from various disciplines contributes to a study that covers a wide variety of topics with relevance for child health. This enables inter- and transdisciplinary research and is likely to generate novel findings. In order to advance the etiologic understanding of complex multi-factorial diseases, the KUNO-Kids study employs modern omics technologies. These provide the opportunity to better characterize both exposures and health outcomes and to consider the dynamics occurring in a life-course perspective.

One major advantage of such a holistic approach incorporating different research questions from different fields of medicine is that non-hypothesis driven but expensive omics technologies must be applied only once in the cohort but the omics data can then be used for many research questions, making the approach cost effective. However, it should be noted that the omics approach goes along with ethical (e.g., data privacy and protection) and scientific challenges—such as the integration of huge amounts of data from different levels or the consideration of omics data in risk assessment and prediction models—and adequate procedures and statistical methods are only now developed [21, 22]. In addition, great emphasis needs to be placed on the pre-analytical sample recovery and work up to ensure a high quality of biomaterial entering these analyses. Thus, tracking of samples and standardized sample procedures are of uttermost importance for the integrity and quality of downstream omics data.

Overall, the socio-demographic characteristics of mothers and fathers in the KUNO-Kids study reflect a population from a prosperous region with good socio-economic living conditions for the majority of its inhabitants. Almost all study participants are employed or were employed before the birth of their child, which is in accordance with the very low rates of unemployment in Germany and especially in the south of Germany. However, the proportion of study participants with a high school leaving certificate is higher than expected, suggesting that such persons are more willing to participate in studies than their less educated counterparts [23]. Census data from the study region show that about 40% of women aged 20 to 39 years obtain a university entrance level (as compared to 56% in our study) [24]. The same applies to the distribution of fathers’ education in the study sample: 34% of men aged 20 to 39 years in the study area obtain a university entrance level [24] as compared to 60% of fathers in the study sample.

The recruitment of a socially diverse sample is a common challenge in population-based research. Migrants or socially disadvantaged people are often underrepresented in epidemiologic cohorts [25, 26]. This is also the case in our study. Previous studies have identified barriers to participation in birth cohorts, such as limited language or literacy skills, burden imposed by excessive information or demanding study procedures, and lack of cultural sensitivity among study personnel [27, 28]. The assessment of reasons for non-participation in the KUNO-Kids study yielded similar findings. In approximately one third of the mothers approached, no informed consent could be achieved due to insufficient German language skills. Many different languages were spoken by the non-German speaking mothers, with no single language spoken by more than 5% of the women. This makes it difficult to design study procedures that would allow integrating non-German speaking families into the study as providing informed consent and understanding the dimension of the cohort study require unambiguous written and oral communication.

On the other hand, experience from other cohorts suggests that at least some barriers to study participation can be overcome by intensified recruitment efforts [27, 29, 30]. Considering the low participation rate in our study, we decided to modify the recruitment strategy in the next phase of the study. By informing and recruiting the mothers already during pregnancy, we acknowledge that some people wish to have more time for their decision about study participation and hope to motivate more families for long-term commitment to the study.

The longer the follow-up period, the more valuable are data derived from birth cohort studies. However, attrition bias caused by loss-to-follow-up is common in longitudinal studies leading to over- or underestimation of effects in case of differential loss-to-follow-up. This also applies to the KUNO-Kids study. The percentage of families who are lost between birth of the child and the 1-year follow-up measure is substantial, and the overrepresentation of well-educated families in the study sample increases over time. This increased selection bias due to attrition does not necessarily affect all exposure-outcomes associations, but it will where loss-to-follow-up is differential. As we saw that study retention is related to socio-economic status, associations between socio-economic status and outcomes [31] will likely be biased. Further, the validity of prevalence and incidence estimates may be compromised. Quantitative bias analysis will be undertaken to estimate the direction, magnitude, and uncertainty arising from systematic errors [32]. For the successful continuation of the study, it will be crucial to ensure that families which remained in the study for the child’s first year of life continue to participate in the future. Measures which are currently implemented to further increase participants’ long-term adherence to the study include the provision of online questionnaires for participants who prefer them over paper-based questionnaires, the partition of questionnaires into smaller portions, and the communication with study participants via regular e-mails with short communications about recent research findings and study news. Inviting participants for clinical follow-up visits in the study center could be a further means for maintaining contact between the study team and participants. These visits are envisioned, but not yet funded, and could be used for performing additional investigations and the validation of specific diagnoses.

Generally, people seem to have become leery with respect to the collection of biomaterial for scientific purposes and not all study participants consent to the collection and processing of biomaterial [33, 34]. In contrast, in the KUNO-Kids cohort, the majority of mothers agreed to the collection and examination of biological samples, including the analysis of genetic information. Against the background of the planned analyses, this high rate of consent is encouraging. It is likely that the hospital setting where both the recruitment of families and the non-invasive collection of biomaterial took place, as well as the above average education levels of the participating parents, the possibility of the continuous consent, and the option of withdrawal at any time contributed to the high acceptance rate. In the first phase of the KUNO-Kids study, the collection of biosamples is restricted to the index child. This limits the scope of research questions which can be addressed. However, many parental biosamples can be acquired at a later time point (e.g., for DNA analysis) when needed for specific research questions where biosamples in informative families can be collected using a case-control design to minimize costs of biomaterial sampling and biobanking. In the next phase of the study, when recruitment takes place in the last trimester of pregnancy, maternal biomaterial (blood, hair, saliva, urine, and microbiome samples) will be collected around delivery.

The KUNO-Kids birth cohort study is embedded in the routines of a maternity and children’s hospital. Therefore, daily health care routines take precedence over study procedures and difficulties in the recruitment process arise from time to time due to incompatibilities between health care and research. However, the study’s embedment in a hospital goes also along with some major advantages: It allows for comprehensive clinical assessments as well as the standardized collection and storage of biosamples—procedures whose implementation is often challenging in large population-based studies. This was achieved by a great effort in standardizing sample procedures going as far as providing ready-to-use sample sets for each biosample and designing digital biosample protocols with our QNOME system (www.qnome.de) which allowed tracking of samples and safely tagging information on sample recovery and storage to each individual sample by using barcodes wherever possible. Further, the study’s integration in a health care setting can help limit the burden imposed on study participants by using existing routine health data (e.g., data from U1 and U2 health check-ups) and avoid additional examinations and assessments. However, this makes it necessary to standardize clinical procedures to study protocols and to perform documentation that exceeds standard clinical practice. Integrating digital devices into clinical practice and standardizing clinical procedures in KUNO-Kids was thus a prerequisite for the use of routine health data. For that purpose, the QNOME system was developed, which facilitates the implementation of digital documentation into clinical procedures. Routine health care data from clinical practice reflects the provision of actual health care under everyday conditions, and access to this data also enables health services research. Finally, the close collaboration between health care providers and researchers represents a starting point for using the birth cohort as a platform for developing and testing interventions in the setting of a cohort of well-informed and well-monitored study participants.

KUNO-Kids is designed as a single-center study. Although the study is based in a hospital where the majority of infants are born in a study region representative for many parts of Germany, generalizability to other populations such as metropolitan regions may be limited. To overcome this potential limitation, efforts were made from the beginning to harmonize assessment methods for exposures and outcomes to allow for a joint analysis with other birth cohorts in Germany.

## Conclusions

Taken together, the data collected in the KUNO-Kids birth cohort study will provide extensive opportunities for investigating child health and its determinants in a holistic approach—in the study area and its adjacent regions as well as in a broader context via data pooling with other birth cohorts. After 3 years of recruitments, 2515 families have been included in the study. This suggests that the minimum aim of 5000 participants is feasible within the scheduled recruitment period of 5 to 10 years. However, like other long-term studies, the KUNO-Kids birth cohort study will need to put effort in continuing to successfully nurture the cohort and maintain participants’ commitment to the study over time. The application of omics data to a wide range of diseases in the same sample bears the potential of identifying novel molecular and mechanistic patterns of health and disease well beyond currently established clinically defined concepts and borders allowing for novel concepts of understanding health and preventing disease development.

## Abbreviations

CSHQ: Childhood Sleep Habits Questionnaire
EBI: Parental Stress Index
F-SozU: Social Support Questionnaire
HLS-EU: European Health Literacy Survey
*M*: Mean
*N*: Number
PSQI: Pittsburgh Sleep Quality Index
SD: Standard deviation
SF-12: Short Form Health Survey
